# Redefining the Treatment Landscape in Gastroparesis: A Clinical Review of Gastric Peroral Endoscopic Myotomy Outcomes and Therapeutic Integration

**DOI:** 10.1002/deo2.70260

**Published:** 2025-12-09

**Authors:** Kobina Essilfie‐Quaye, Carson Creamer, Mohammad Abuassi, Harsimran Kalsi, Neeka Akhavan, Tony Brar, Yaseen Perbtani

**Affiliations:** ^1^ University of Central Florida College of Medicine Orlando Florida USA; ^2^ Department of Internal Medicine HCA Florida North Florida Hospital Gainesville Florida USA; ^3^ Division of General Internal Medicine University of Florida Gainesville Florida USA; ^4^ Center For Advanced Therapeutic Endoscopy (CATE) HCA Florida North Florida Hospital Gainesville Florida USA; ^5^ Digestive Disease Associates Gainesville Florida USA

**Keywords:** EndoFLIP, endoscopic myotomy, gastroparesis, G‐POEM, pyloric dysfunction

## Abstract

Gastroparesis is a chronic gastric motility disorder marked by delayed gastric emptying in the absence of mechanical obstruction. For patients with symptoms refractory to medical and dietary therapy, Gastric Peroral Endoscopic Myotomy (G‐POEM) has emerged as a minimally invasive, pylorus‐targeted intervention that directly addresses pyloric dysfunction.

Technical success rates consistently exceed 95%, reflecting procedural standardization and a favorable learning curve. Short‐term symptom improvement is reported in up to 80% of patients and is often accompanied by enhanced gastric emptying and quality of life (QOL). Long‐term outcomes show sustained clinical success of 50%–77.5% at 3–4 years, influenced by disease etiology and neuromuscular progression. Endoluminal functional lumen imaging probe, a functional imaging tool that measures pyloric distensibility, has shown promise in identifying optimal candidates and assessing procedural efficacy, though standardized predictive thresholds are still lacking.

Overall, G‐POEM has become a leading pylorus‐directed therapy for refractory gastroparesis. Comparative data suggest that G‐POEM offers superior clinical efficacy and durability relative to botulinum toxin injection and gastric electrical stimulation, with fewer complications than surgical pyloroplasty.

This review integrates the current clinical evidence supporting G‐POEM, with a focus on patient selection, procedural outcomes, QOL, and durability of response. It also examines comparative data with alternative therapies and addresses ongoing areas of controversy, including procedural technique, repeat intervention, and the predictive value of prior pyloric‐directed therapies. G‐POEM represents a safe and effective treatment modality for appropriately selected patients and continues to shape the landscape of gastroparesis management.

## Graphical abstract

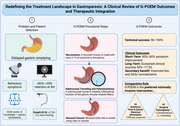

**Abbreviations**
ACG: American College of Gastroenterology
AGA: American Gastroenterological Association
DI: distensibility index
EndoFLIP: endoluminal functional lumen imaging probe
GCSI: gastroparesis cardinal symptom index
GES: gastric electrical stimulation
GESc: gastric emptying scintigraphy
GI: gastrointestinal
GIQLI: gastrointestinal quality of life index
G‐POEM: gastric peroral endoscopic myotomy
OR: odds ratio
POEM: peroral endoscopic myotomy
QOL: quality of life
SDD: same‐day discharge
SF‐36: Short Form‐36

## 1 Introduction

Gastroparesis is a chronic disorder of gastrointestinal (GI) motility, marked by delayed stomach emptying despite the absence of mechanical obstruction [1]. Patients commonly experience debilitating symptoms, including nausea, vomiting, postprandial fullness, early satiety, and abdominal pain, often exacerbated by meals. The burden of gastroparesis extends beyond physical symptoms, significantly impairing quality of life (QOL) and increasing healthcare utilization [2]. Recent epidemiological data indicate a rising prevalence of gastroparesis in the United States, with the estimated incidence ranging from 13.8 to 267.7 per 100,000 adults [3]. Women are disproportionately affected, nearly four times more likely to be diagnosed with gastroparesis than men [4]. While the etiology in most patients remains elusive, diabetes, post‐surgical changes, and medication‐induced effects are the most common contributors [5].

The pathophysiology is multifactorial, involving a complex interplay of neuromuscular dysfunction, pyloric dysmotility, chronic low‐grade inflammation, and disruptions in neurohormonal signaling [4]. Impaired vagal nerve signaling, dysfunction of the interstitial cells of Cajal, and pyloric hypertonicity contribute to delayed gastric emptying [1]. Moreover, increasing evidence highlights that pyloric dysfunction is a critical therapeutic target, paving the way for the development of pyloric‐directed interventions [1]. Treatment strategies for gastroparesis have traditionally centered on dietary modifications and pharmacological agents, including metoclopramide, domperidone, and erythromycin [6]. Non‐pharmacological interventions, such as gastric electrical stimulation (GES) and surgical pyloroplasty, are typically reserved for refractory cases. However, their use is limited by invasiveness, variable efficacy, and inconsistent long‐term outcomes [7]. Among emerging therapies, Gastric Peroral Endoscopic Myotomy (G‐POEM) has gained recognition as a minimally invasive, pyloric‐directed technique offering symptom relief and improved gastric emptying in refractory cases [8].

Since its first introduction [9, 10], G‐POEM has altered the therapeutic landscape for gastroparesis. By targeting pyloric dysfunction through endoscopic myotomy, G‐POEM improves gastric emptying and alleviates symptoms in patients unresponsive to conventional treatments. Recent clinical studies have demonstrated promising short‐ and long‐term outcomes, with clinical success rates reaching ∼80% at 6 months in selected populations, prompting recommendations from major gastroenterology societies for its use in refractory gastroparesis cases [7, 11]. This review explores the evolution of G‐POEM, its procedural technique, clinical outcomes, safety profile, and predictors of success. Additionally, we compare G‐POEM with alternative treatment modalities and discuss future directions for improving its role in gastroparesis management.

## 2 Patient Selection for G‐POEM

### 2.1 General Indications and Clinical Considerations

G‐POEM is typically reserved for patients with refractory gastroparesis unresponsive to dietary modifications and pharmacologic therapy [7]. It has demonstrated clinical benefit in patients with diabetic, idiopathic, and post‐surgical etiologies of gastroparesis [12]. Conversely, the American Gastroenterological Association (AGA) Clinical Practice Update recommends against offering G‐POEM to most patients with post‐infectious gastroparesis, given their less predictable and transient disease course [13].

While some patients may have previously undergone alternative therapies such as botulinum toxin injection, transpyloric stenting, or GES, failure of these modalities is not required before considering G‐POEM. Additionally, prior response or lack thereof to these treatments does not reliably predict procedural success [13].

### 2.2 Addressing Opioid‐Induced Gastroparesis

Chronic opioid use significantly impacts GI motility. By inhibiting neurotransmitter release from enteric neurons, opioids reduce nitrergic and cholinergic activity essential for pyloric relaxation and antral contraction, producing persistent pyloric spasm and impaired gastric emptying. Consequently, opioids can induce persistent pyloric spasm, impairing coordinated gastric emptying [14]. Patients on chronic opioids typically exhibit more severe gastroparesis symptoms, increased healthcare utilization, and poorer QOL than opioid‐naïve individuals. Importantly, opioid‐related symptoms may persist even after procedures targeting pyloric dysfunction, due to continued opioid‐induced inhibition of pyloric relaxation and antral motility [14].

Current expert consensus strongly recommends opioid weaning whenever feasible prior to G‐POEM, with reassessment of gastric emptying 48 h after cessation to confirm persistent gastroparesis [13]. However, opioid discontinuation may not always be achievable. In such cases, peripherally acting μ‐opioid receptor antagonists (PAMORAs) such as methylnaltrexone, naloxegol, and alvimopan have been investigated as potential means to mitigate opioid‐related gastroparesis. While these agents effectively treat opioid‐induced constipation, studies in opioid‐naïve participants have shown that standard‐dose PAMORAs do not reverse opioid‐induced delays in gastric emptying [15, 16, 17]. Although data on gastroparesis are limited, they may warrant future study as adjunctive therapy in patients unable to discontinue opioids.

Recent data from a multicenter study indicate that G‐POEM can still provide symptomatic relief for patients unable to discontinue opioids, achieving a clinical response rate of approximately 60.5%. Responders also exhibited significantly lower baseline pyloric distensibility than non‐responders, suggesting pyloric compliance may predict outcomes in this subgroup [18]. Thus, while opioid weaning remains ideal, G‐POEM represents a viable compassionate option for patients who must continue opioid therapy.

### 2.3 Diagnostic Criteria for Procedural Consideration

According to guidelines from the American College of Gastroenterology (ACG), adult patients may be considered for G‐POEM if the following diagnostic criteria are met [7].

- Mechanical obstruction is excluded, typically via upper endoscopy or contrast imaging.
- Delayed gastric emptying is documented by solid‐phase gastric emptying scintigraphy (GESc), defined as >10% gastric retention at 4 h using a standardized low‐fat egg‐white meal. Importantly, a retention threshold greater than 20% at 4 h has been associated with an increased likelihood of response to G‐POEM.
- Moderate to severe symptoms are present, predominantly nausea and vomiting, which predict favorable outcomes. Patients with pain‐predominant symptoms are less likely to improve following G‐POEM.

### 2.4 Symptom Severity Assessment Using the Gastroparesis Cardinal Symptom Index

The gastroparesis cardinal symptom index (GCSI) is a validated, patient‐reported scoring tool used to quantify symptom severity in gastroparesis [19]. The GCSI assesses nine symptoms grouped into three domains: nausea/vomiting, postprandial fullness/early satiety, and bloating. Patients rate symptom severity on a 0–5 Likert scale, with the total score calculated either as the average score of all items (0–5 scale) or as a summative total (0–45 scale), with higher scores indicating greater severity.

Baseline GCSI scores have predictive value regarding response to pyloric‐directed therapies like G‐POEM. Studies suggest that patients with moderate‐to‐severe baseline GCSI scores (>2.6 on the 0–5 scale) have a higher odds ratio (OR ∼3.2) of achieving clinical success at 12 months [20]. Symptom patterns also matter; patients whose predominant symptoms are postprandial fullness and early satiety tend to benefit most, whereas those with pain predominant symptoms respond less favorably [13].

### 2.5 Role of Endoluminal Functional Lumen Imaging Probe in Evaluating Patients for G‐POEM

Endoluminal functional lumen imaging probe (EndoFLIP) is an advanced diagnostic tool that uses impedance planimetry to objectively assess the geometry and distensibility of GI sphincters [21]. In gastroparesis, EndoFLIP measures pyloric cross‐sectional area (CSA) and distensibility index (DI), parameters reflecting pyloric compliance. This is clinically relevant for G‐POEM, as a dysfunctional, stiff pylorus often contributes to gastroparesis symptoms. Previous prospective studies have suggested selecting patients for G‐POEM who exhibit low baseline pyloric DI (<9.2 mm^2^/mmHg at 50 mL balloon fill), reflecting significant pyloric dysfunction and potential benefit from myotomy [22]. A recent prospective study by Farooq et al. further refined this criterion, demonstrating that a DI threshold ≤7.35 mm^2^/mmHg significantly predicted clinical response (specificity 80.8%, positive predictive value 88.1%) [23]. Furthermore, sustained improvements in pyloric distensibility at 6 months correlated strongly with clinical success, whereas distensibility gains that diminished over time were typical of nonresponders. These threshold parameters and related selection criteria are summarized in Table 1.

**TABLE 1:** Summary of ideal gastric peroral endoscopic myotomy (G‐POEM) candidate criteria.

Parameter	Preferred threshold	Notes
GESc at 4 h	>20% retention	Predictor of improved clinical outcome [20]
GCSI Total Score	>2	Suggests moderate‐to‐severe symptoms [20, 62]
Dominant Symptoms	Nausea/vomiting	Strongest correlation with success [13]
Etiology	Diabetic, idiopathic, post‐surgical	Avoid in post‐infectious cases [13]
EndoFLIP DI	7.35–9.2 mm^2^/mmHg^a^	Optional physiologic predictor [22, 23]
Prior Therapy	Not required	Botox, stents, or GES failure not necessary [13]
Opioid Use	Off opioids + retested GESc	Avoid premature intervention in reversible cases [13]
Predominant Pain	Relative contraindication	Poor response to pyloric therapy [13]

^a^ DI threshold of ≤7.35 mm^2^/mmHg has recently been validated, while earlier studies suggested a threshold of <9.2 mm^2^/mmHg. GESc, Gastric Emptying Scintigraphy; GCSI, Gastroparesis Cardinal Symptom Index; DI, Distensibility Index; EndoFLIP, Endoluminal Functional Lumen Imaging Probe; GES, Gastric Electrical Stimulation.

Despite these promising findings, a 2024 systematic review reported inconsistent results across studies, and a universally validated prognostic model for EndoFLIP remains unavailable [24]. These differences likely stem from the dynamic nature of pyloric physiology, heterogeneity in measurement techniques, and limited sample sizes. Therefore, EndoFLIP thresholds should be viewed as guidance rather than definitive predictors of response. In current practice, EndoFLIP is best applied to confirm pyloric dysfunction before G‐POEM and to assess myotomy adequacy intra‐procedurally. Future research should prioritize standardizing acquisition techniques, integrating EndoFLIP with complementary physiologic tests, and conducting large prospective multicenter trials to validate predictive thresholds and improve clinical reliability.

### 2.6 Patient Preparation and Peri‐Procedural Considerations

Proper patient preparation is essential to ensure the safety and effectiveness of G‐POEM. Patients are typically placed on a clear‐liquid diet for 1–3 days followed by overnight fasting (8–12 h) to minimize gastric residual volume, enhance visualization, and reduce aspiration risk [13].

Given the transmural nature of the gastric incision in G‐POEM, routine periprocedural antibiotic prophylaxis is recommended [25]. While no standard regimen exists, broad‐spectrum antibiotics such as piperacillin‐tazobactam or levofloxacin are commonly used to cover gram‐negative and anaerobic organisms. Although evidence supporting antibiotic prophylaxis is limited, prevailing practice guidelines advocate its use to minimize potential infections.

To further ensure patient safety and procedural efficacy, G‐POEM is performed under general anesthesia with endotracheal intubation to protect the airway and prevent aspiration, while ensuring immobility during endoscopic myotomy [26]. Neuromuscular blockade further facilitates precise dissection by eliminating movement. Continuous monitoring of peak airway pressures is also critical, as elevations may indicate excessive gastric insufflation or pneumoperitoneum. Anesthesia providers generally maintain pressures below 20 cmH_2_O and adjust ventilation as needed to prevent respiratory compromise [26].

### 2.7 G‐POEM Step‐by‐Step Technique

**FIGURE 1:**
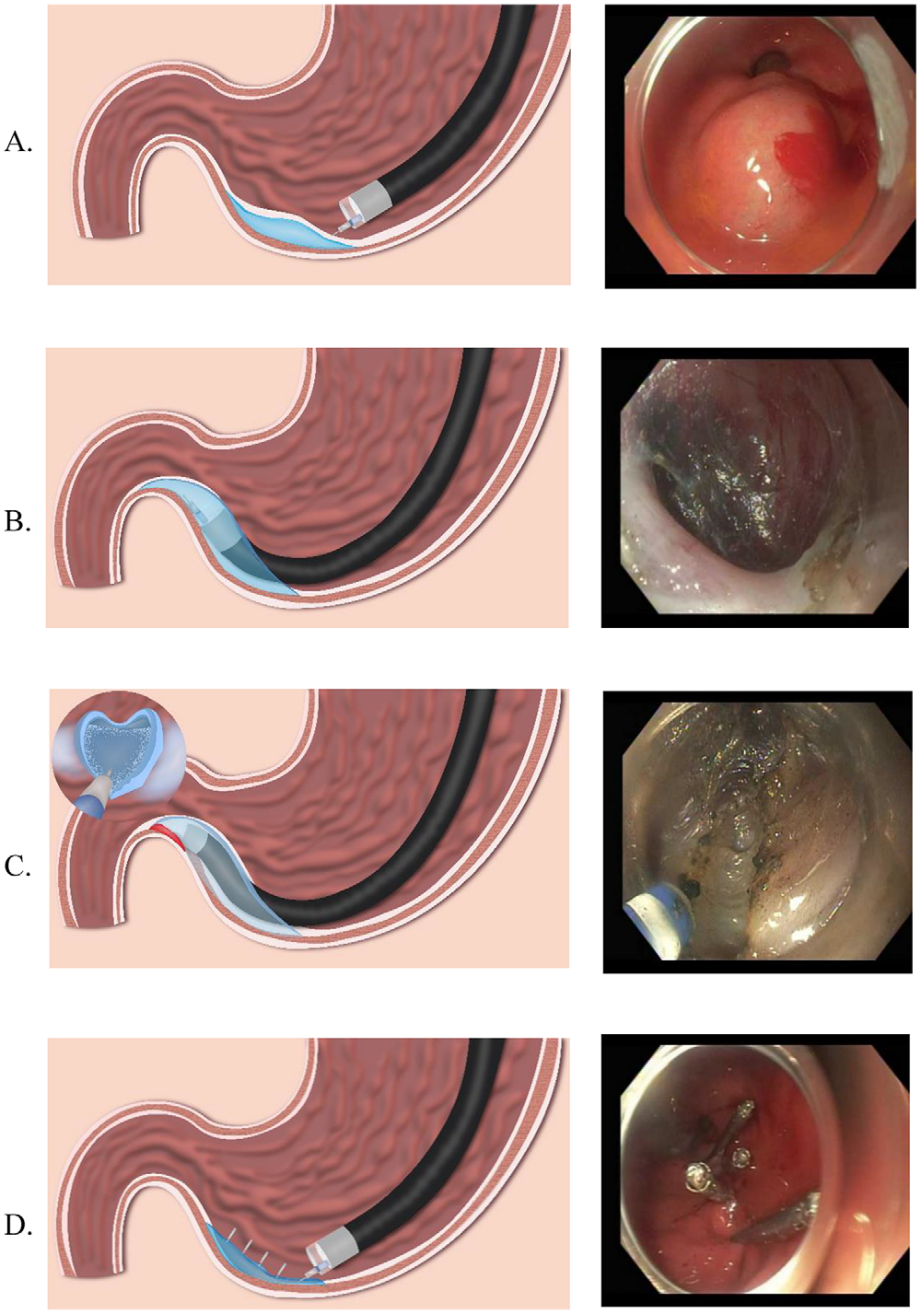
Procedural steps of gastric peroral endoscopic myotomy (G‐POEM). (A) Step 1: Submucosal injection. Initial mucosal entry was made approximately 4–5 cm proximal to the pylorus along the greater curvature. (B) Step 2: Tunneling. Creation of a submucosal tunnel directed toward the pyloric sphincter. (C) Step 3: Pyloromyotomy. Myotomy of the pyloric sphincter using a Hybrid I‐type knife. (D) Step 4: Mucosal closure. Closure of the mucosal incision using endoscopic clips.

G‐POEM is performed using a high‐frequency electrosurgical generator (ERBE VIO 300D or VIO 3; Erbe Elektromedizin, Tübingen, Germany) in combination with endoscopic knives such as the Triangle Tip Knife, DualKnife or DualKnife J, HookKnife, IT Knife (Olympus Medical Systems, Tokyo, Japan), or the HybridKnife (ERBE Elektromedizin, Tübingen, Germany). The choice of knife depends on operator preference and case complexity, as each allows precise cutting and controlled coagulation, with some devices offering built‐in fluid injection capability. The procedure involves four sequential steps: mucosotomy, submucosal tunneling, pyloromyotomy, and closure, as illustrated in Figure 1.

### 2.8 Mucosotomy

The endoscope is equipped with a clear distal attachment cap to enhance stability and visualization during mucosal incision and tunneling. The mucosotomy typically measures 1.5–2 cm and may be oriented longitudinally or transversely [13]; in our practice, a transverse incision is favored for ease of closure. Mucosal entry is generally created using the DualKnife J or HybridKnife with an electrosurgical generator set to EndoCut Q mode (effect 2–3:1:1) or Dry Cut mode (50 W, effect 3) to achieve a controlled incision.

The mucosal entry point is typically created on the gastric antrum, proximal to the pylorus, as previously described [10, 27]. Traditionally, the entry point was placed 4–5 cm proximal to the pylorus on the greater curvature side. Increasingly, operators favor creating the mucosotomy closer to the pylorus, approximately 2–3 cm proximal, to minimize unnecessary submucosal tunneling while maintaining sufficient safety margins. A lesser curvature approach may also be employed, offering reduced looping and fluid pooling [13, 28]. Recent comparative data have shown no significant difference in technical or clinical success rates between the two approaches, supporting a tailored, anatomy‐based strategy [29].

### 2.9 Submucosal Tunneling

After mucosal incision, the submucosal space is achieved by injecting a lifting solution (normal saline or hydroxyethyl starch with indigo carmine or methylene blue). Epinephrine (1:10,000 dilution) may be added to minimize bleeding. Repeat injections are generally required during tunneling to maintain submucosal elevation. Tunneling is advanced toward the pylorus using an electrocautery knife, typically in Swift Coagulation (effect 3–4, 40–60 W) or Spray Coagulation (∼35–50 W) mode for controlled submucosal dissection, with Soft Coagulation (∼80 W, effect 5) applied for targeted hemostasis as needed.

The pyloric ring appears as a distinct, thick, whitish circular band, clearly contrasting with the thinner and looser duodenal submucosa beyond it. Additionally, tactile feedback indicating resistance or narrowing helps confirm the pyloric location. The tunnel is extended ∼1 cm beyond the pyloric ring into the duodenal bulb, avoiding excessive tunneling beyond this point due to the thin and vascular duodenal wall. Advanced localization techniques, such as the navigational tunnel method, may be employed to streamline direct identification of the pylorus, thus minimizing unnecessary tissue dissection [30].

### 2.10 Pyloromyotomy

Once the pyloric ring is exposed within the submucosal tunnel, a myotomy is performed, typically extending ∼2 cm proximally into the gastric antrum. Myotomy is generally performed using EndoCut Q mode (effect 2–3:1:1) or Dry Cut mode (50–80 W, effect 3) for precise division of the circular fibers. At a minimum, the inner circular muscle layer of the muscularis propria should be incised. However, performing a full‐thickness pyloromyotomy through the circular muscle fibers, extending to, but not breaching, the outer longitudinal muscle and subserosal layer, is advisable to minimize risks such as capnoperitoneum, vessel injury, and postprocedural pain related to serosal injury [13]. A standard electrocautery knife may be exchanged for an insulated‐tip knife to further reduce the risk of mucosal injury to the pylorus and duodenal bulb, although this is optional. Myotomy can also be effectively performed using a needle‐type knife. Evidence comparing partial (circular only) versus full‐thickness pyloromyotomy remains limited. Similarly, the benefit of wider “double” or wedge myotomy over the conventional approach is not yet clearly established. A recent comparative study suggested short‐term symptom improvement with a double myotomy, but larger prospective trials are needed to confirm its significance and durability [31].

### 2.11 Closure

Secure closure of the mucosal entry site is essential to prevent leakage and related complications. Through‐the‐scope clips are most commonly employed for closure due to simplicity, availability, and proven efficacy. Endoscopic suturing systems (X‐Tack, OverStitch) serve as alternatives for large or fibrotic defects. A recent meta‐analysis found comparable success between clipping (94.4%) and suturing (100%) with similarly low complication rates [32]. Although suturing systems have higher per‐device costs, overall procedural expenses are comparable to clipping. Ultimately, the closure method should be based on defect size, operator expertise, and device availability.

### 2.12 Post‐Procedural Recovery

Following G‐POEM, patients are monitored in the post‐anesthesia care unit. Same‐day discharge (SDD) is safe and feasible in carefully selected patients without complications, significant symptoms, or social barriers [33, 34]. Patients discharged on the same day should receive follow‐up within 48 h to screen for potential adverse events. There is no clear consensus on routine post‐procedural imaging, such as contrast esophagram, following G‐POEM [34]. However, selective imaging should be considered in cases involving challenging mucosal closures or new concerning symptoms suggestive of a leak. Diet typically advances from clear liquids to regular foods over 1–2 weeks, guided by tolerance and clinical judgment.

### 2.13 Adverse Events

G‐POEM is a minimally invasive therapy with an overall favorable safety profile and no reported procedure‐related mortality [35, 36, 37]. Bleeding occurs in approximately 4.8% of procedures [37], though severe hemorrhage remains infrequent compared to surgical alternatives [35]. Despite involving mucosal incision and tunneling, the incidence of clinically significant perforation or leakage is low (∼1%–2%) [37], largely due to meticulous endoscopic closure techniques. Its incisionless approach also eliminates external wound infections, while systemic infections are uncommon, occurring in ∼0.3% of cases [37]. Post‐procedure, patients typically experience mild abdominal discomfort [35], but recovery is rapid, with many resuming a regular diet shortly after the procedure and qualifying for SDD [34].

Although bile or duodenogastric reflux has been raised as a potential concern following pylorus‐modifying procedures, current evidence suggests these events are uncommon after G‐POEM. Computational modeling suggests that excessive pyloric enlargement may permit limited retrograde flow [38], yet clinical data show this to be rare. A 2025 systematic review reported bile acid gastritis rates of 0%–15.4% across studies of pyloroplasty and G‐POEM, though interpretation is limited by small sample sizes and heterogeneous follow‐up [39]. Nonetheless, further prospective studies are warranted to better define the true incidence and clinical relevance of this phenomenon.

## 3 Clinical Outcomes of G‐POEM

### 3.1 Technical Success

Technical success, defined as successful completion of submucosal tunneling, pyloromyotomy, and mucosal closure, remains consistently high at 96%–100% [40, 41, 42]. While most reports originate from high‐volume expert centers, a learning curve analysis by Reja et al. demonstrated that procedural efficiency is typically achieved after approximately 18 cases, suggesting that proficiency can be attained early in clinical practice [43]. Together with multiple single‐center reports of 100% technical success [42, 44, 45, 46] and pooled analyses encompassing more than 500 procedures [40, 41], these findings support the technical reliability and reproducibility of G‐POEM across diverse practice settings.

### 3.2 Short‐Term Outcomes

G‐POEM has consistently demonstrated significant clinical improvement within 6 months of the procedure. Across multiple prospective studies, 60% to 80% of patients experience meaningful symptom relief, typically measured by the GCSI [20, 35, 40, 47]. Clinical success is commonly defined as at least a 1‐point decrease in the total GCSI score with a ≥25% improvement in two or more GCSI subdomains. Mean GCSI scores typically improve from ∼3.3 at baseline to about 1.4–1.6 by 6 months, with notable symptom relief observed as early as 1 month post‐procedure [20]. Rapid improvements typically include reductions in nausea, early satiety, bloating, and postprandial fullness. Concurrent physiological improvement is often demonstrated by GESc, with normalization occurring in 40%–70% of patients within the same period [12, 20]. Further high‐quality evidence supports G‐POEM's efficacy, including a pivotal sham‐controlled randomized trial by Martinek et al., which reported a 71% clinical response rate at 6 months compared to 22% in the sham group (*p* = 0.005) [47]. This randomized trial corroborates prior findings from largely non‐controlled studies.

### 3.3 Long‐Term Outcomes

Long‐term data from multicenter cohorts confirm that the therapeutic benefits of G‐POEM extend up to 3–4 years of follow‐up. Sustained clinical response rates range from 56%–70% at 1 year and approximately 50%–77.5% at 2–4 years [20, 28, 48, 49, 50]. Meta‐analyses report pooled success rates of ∼75% at 3 years [49]. In the largest prospective cohort, Hernández Mondragón et al. demonstrated a 77.5% response at 24 months, particularly durable in diabetic patients [28]. Labonde et al. reported 65.2% success at 3 years, while Abdelfatah et al. found 69.1% success at 1 year with an estimated recurrence of ∼13% annually [48, 50].

Variability in long‐term efficacy likely reflects progression of the underlying neuromuscular disorder and fibrotic remodeling at the pylorus, rather than procedural failure. Rare cases of incomplete myotomy may also contribute to early recurrence but remain mechanistically distinct from disease progression. Future studies integrating physiologic assessment tools such as EndoFLIP and gastric motility mapping may help clarify these mechanisms and optimize long‐term outcomes. Long‐term data are summarized in Table 2.

**TABLE 2:** Clinical outcomes of gastric peroral endoscopic myotomy (G‐POEM) by etiology.

Etiology	Clinical success (%)^*^	GESc normalization (%)^*^	References
Idiopathic	∼60%–75%	∼61.7% [28]	[28, 35, 44, 47]
Diabetic	∼65%–90%	∼69.5% [28]	[28, 35, 44, 47]
Postsurgical	∼50%–70%	∼58.8% [28]	[28, 35, 44, 47]

* Reported ranges reflect variability across studies with differing sample sizes, patient demographics, and follow‐up durations (ranging from 6 months to 48 months), which may influence clinical success and GESc normalization rates.

### 3.4 Comparative Role of G‐POEM within the Therapeutic Algorithm

When evaluated alongside alternative modalities, G‐POEM has emerged as a pivotal therapy for refractory gastroparesis. Pharmacologic agents remain first‐line, yet efficacy is modest, and tachyphylaxis limits durability [7]. Botulinum toxin injection provides only a transient benefit without superiority over placebo in randomized trials [51, 52, 53]. GES may help selected patients with predominant nausea and vomiting, but outcomes remain inconsistent, and its use is limited to humanitarian device exemption [54, 55, 56, 57]. Surgical pyloroplasty achieves similar efficacy but with greater morbidity and recovery time, and is increasingly being replaced by endoscopic pyloromyotomy [58, 59, 60].

G‐POEM offers >95% technical success, durable symptom relief in 50%–77.5% at 2–4 years, and a favorable safety profile. The 2022 ACG guideline supports its use in medically refractory disease, while the current 2025 AGA guideline emphasizes careful patient selection and shared decision‐making, underscoring G‐POEM's role as a targeted option for those failing conventional therapy [7, 61].

**FIGURE 2:**
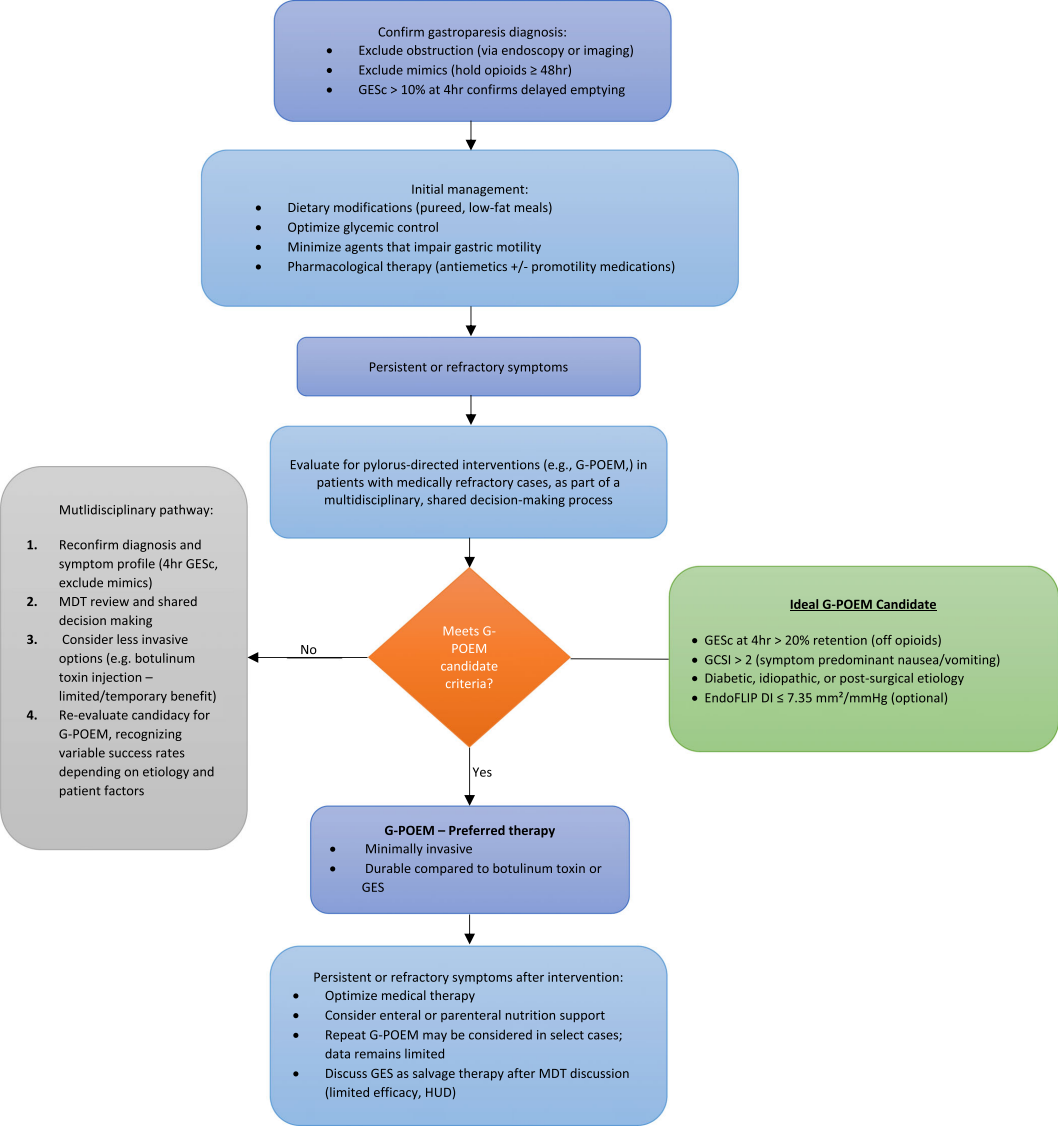
Proposed treatment algorithm for gastroparesis. Following confirmation of delayed gastric emptying, patients progress from dietary and medical optimization to pylorus‐directed interventions as indicated. G‐POEM is the preferred minimally invasive option, with multidisciplinary evaluation guiding management of refractory cases. Adapted in part from the AGA Clinical Practice Guideline on Gastroparesis (2025) and modified by the authors to include additional management considerations. GESc, gastric emptying scintigraphy; GES, gastric electrical stimulation; G‐POEM, gastric peroral endoscopic myotomy; GCSI, Gastroparesis Cardinal Symptom Index; MDT, multidisciplinary team; DI, distensibility index (from EndoFLIP); HUD, Humanitarian Use Device.

Comparative outcomes and a proposed treatment algorithm, developed to complement existing AGA and ACG guidance, are summarized in Table 3 and Figure 2.

**TABLE 3:** Comparative overview of therapies for refractory gastroparesis.

Therapy	Mechanism	Clinical response	Effect on GESc normalization^a^	Effect on QOL^b^	Adverse events	References
G‐POEM	Endoscopic pyloric myotomy	60%–80% (6–12 mo), ∼48%–77.5% (≥12 mo)	Moderate to high	Improved SF‐36 in ∼65%–75% (≤12 mo), ∼45%–55% (>12 mo)	Mild transient abdominal pain (∼7%) Minor bleeding (∼5%) Rare systemic infection (<1%) Total complications ∼12.5%, no severe events	[20, 28, 35, 40, 44, 47, 48, 49, 50]
Surgical Pyloroplasty	Surgical pyloromyotomy	70%–90% (6–34 mo)	Moderate to high	Limited formal QOL data	Infection (∼2%) Leak (∼1%) Significant bleeding (∼1%) Readmission (∼7%) Total complications ∼23%	[37, 58, 59, 60]
Gastric electrical stimulation	Antral electrical stimulation	30%–50%	Minimal	Improved SF‐36; inconsistent GIQLI.	Infection (∼6%–9%) Lead dislodgement (∼2%) Abdominal wall pain (∼16%)	[54, 55, 56, 57]
Botulinum toxin	Chemical denervation	30%–40% (short‐term)	Minimal	Limited improvement; GIQLI improvement mainly if low pyloric distensibility	Headaches (∼16%) Fatigue (∼19%) No severe events	[51, 52, 53, 68]
Medical therapy	Pharmacologic prokinetics	20%–60% (varies by agent)	Minimal	Modest improvement (PAGI‐QOL); limited data otherwise	QT prolongation/arrhythmias Extrapyramidal effects GI distress (N/V/D) Tachyphylaxis	[7, 8]

Abbreviations: GESc, gastric emptying scintigraphy; GES, gastric electrical stimulation; G‐POEM, gastric peroral endoscopic myotomy; mo, months; SF‐36: Short Form Health Survey (general health); GIQLI, Gastrointestinal Quality of Life Index; PAGI‐QOL: Patient Assessment of Upper Gastrointestinal Disorders Quality of Life.

^a^ GESc Normalization Definitions: Moderate to High: 40%–70% normalization; Moderate: 20%–40% normalization; Minimal: <20% normalization.

^b^ Improvement in QOL Definition: Defined as ≥7–10‐point gains in SF‐36 domains, and ≥10–14‐point increase in GIQLI.

*Note*: Clinical response reflects overall reported rates from published short‐ and long‐term studies. G‐POEM demonstrates a generally favorable safety profile compared to surgical alternatives.

### 3.5 Quality of Life

Beyond symptomatic relief and improved gastric motility, G‐POEM has consistently been associated with meaningful improvements in patient QOL. Several prospective studies have reported significant gains in Short Form‐36 (SF‐36) Health Survey domain scores and GCSI metrics, particularly in physical functioning, vitality, and mental health [12, 62]. These QOL improvements often emerge within the first month after the procedure and persist for at least 6–12 months. Additionally, enhancements in nutritional status, oral intake, and reduced emergency department visits further highlight the broad clinical impact of G‐POEM [12, 62].

## 4 G‐POEM in Refractory Gastroparesis: Special Populations and Technique Considerations

### 4.1 G‐POEM in Patients With Prior Pyloric Interventions

Patients with refractory gastroparesis may experience symptom relapse following initial G‐POEM, prompting consideration of redo G‐POEM. In a large retrospective series, 69% maintained clinical response at 1 year, with an estimated annual response loss of ∼13%, indicating a subset prone to recurrence [50]. Recent data indicate that 6‐month clinical success is comparable between prior pyloric intervention and primary G‐POEM, at approximately 50% and 56%, respectively [63].

Redo G‐POEM is technically feasible but often more challenging due to submucosal fibrosis from prior interventions. Symptom recurrence may result from incomplete myotomy, fibrotic remodeling, recurrent pyloric hypertonicity, or progression of the underlying neuropathic disorder. Patient‐related factors such as longer disease duration, higher BMI, psychiatric medication use, and opioid exposure have also been associated with reduced durability and higher recurrence risk [50, 64]. When re‐intervention is required, shifting from a greater to a lesser curvature approach may facilitate a more complete myotomy and improve technical success [13].

For patients with previous surgical pyloromyotomy, G‐POEM remains technically achievable but involves heightened complexity due to scar tissue and fibrosis from prior surgery. In these cases, a greater curvature approach is generally advisable. Given the limited direct data on G‐POEM post‐surgical pyloromyotomy, meticulous patient selection and thorough pre‐procedural planning are essential. Further prospective studies are needed to establish clear guidelines and optimal management strategies for these complex clinical scenarios.

### 4.2 Combined G‐POEM With GES

The integration of G‐POEM with GES represents an innovative therapeutic approach for refractory gastroparesis. G‐POEM targets mechanical pyloric resistance, while GES modulates gastric neuromuscular function electrically [13, 65]. In a recent study, patients experiencing persistent symptoms following GES underwent G‐POEM as rescue treatment, and outcomes were compared with those undergoing G‐POEM alone. Clinical success was similar between both groups (∼66% for combined GES and G‐POEM versus ∼65% for G‐POEM alone), with similar complication rates [66]. Although no definitive synergistic effect was identified, G‐POEM appears safe and effective in patients with prior GES, offering an additional option for treatment‐refractory patients.

### 4.3 G‐POEM in Patients With Altered Gastric Anatomy

Previous gastric surgeries such as fundoplication, sleeve gastrectomy, or esophagectomy can cause gastroparesis through disrupted gastric motility, vagal nerve injury, or altered compliance [6]. Despite these technical complexities, G‐POEM has shown high technical success (∼100%) and a favorable safety profile in these patients [67]. A recent systematic review reported a pooled clinical success rate of ∼70% in postsurgical gastroparesis, comparable to results in diabetic and idiopathic cases [35]. Procedural adjustments, such as navigating around surgical scar tissue or utilizing alternative anatomical approaches, enhance feasibility and outcomes. Thus, G‐POEM represents a safe and effective therapeutic option for refractory gastroparesis following gastric surgery, provided careful patient selection and individualized technical strategies are employed.

## 5 Conclusion

G‐POEM has advanced the management of refractory gastroparesis, providing a minimally invasive treatment with high technical success and durable relief. Comparative studies consistently demonstrate favorable efficacy and safety compared to alternative therapies. While careful patient selection remains essential, growing evidence supports its use across a broad spectrum of patients. Future research should focus on standardizing procedural techniques, establishing reliable predictors of response, and conducting prospective multicenter studies with extended follow‐up. These efforts are essential to clarify mechanisms of symptom recurrence, such as disease progression, fibrosis, or incomplete myotomy, and to better define the role of redo G‐POEM in recurrent disease.

## Author Contributions

**Tony Brar** and **Yaseen Perbtani**: Conceptualized the review and supervised the project. **Kobina Essilfie‐Quaye**, **Carson Creamer**, **Mohammad Abuassi**, and **Harsimran Kalsi**: Drafted the manuscript and contributed to subsequent revisions. **Kobina Essilfie‐Quaye** and **Carson Creamer**: Designed and created G‐POEM cartoon images and a graphical abstract. **Neeka Akhavan**, **Tony Brar**, and **Yaseen Perbtani**: Provided critical review, mentorship, and senior oversight. All authors: Reviewed and revised the manuscript and approved the final version for publication.

## Funding

No funding was received for this study.

## Conflicts of Interest

The authors declare no conflicts of interest.

## Ethics Statement

This review article did not involve original research with human participants and therefore did not require Institutional Review Board approval. All clinical images used are de‐identified and do not contain any patient‐identifiable information.
